# Glycoengineering Human Neural and Adipose Stem Cells with Novel Thiol-Modified *N*-Acetylmannosamine (ManNAc) Analogs

**DOI:** 10.3390/cells10020377

**Published:** 2021-02-12

**Authors:** Jian Du, Christian Agatemor, Christopher T. Saeui, Rahul Bhattacharya, Xiaofeng Jia, Kevin J. Yarema

**Affiliations:** 1 Department of Neurosurgery, University of Maryland School of Medicine, Baltimore, MD 21201, USA; jdu@som.umaryland.edu; 2 Department of Biomedical Engineering, The Johns Hopkins School of Medicine, Baltimore, MD 21205, USA; cagatem1@jhmi.edu (C.A.); chris.saeui@gmail.com (C.T.S.); rahul.chemiit@gmail.com (R.B.); 3 Translational Cell and Tissue Engineering Center, The Johns Hopkins School of Medicine, Baltimore, MD 21231, USA; 4 Department of Orthopedics, University of Maryland School of Medicine, Baltimore, MD 21201, USA; 5 Department of Anatomy and Neurobiology, University of Maryland School of Medicine, Baltimore, MD 21201, USA; 6 Department of Anesthesiology and Critical Care Medicine, The Johns Hopkins School of Medicine, Baltimore, MD 21205, USA; 7 Department of Chemical and Biomolecular Engineering, The Johns Hopkins University, Baltimore, MD 21218, USA; 8 The Sidney Kimmel Comprehensive Cancer Center, Department of Oncology, The Johns Hopkins School of Medicine, Baltimore, MD 21231, USA

**Keywords:** metabolic glycoengineering, *N*-acetylmannosamine (ManNAc) analogs, neural stem cell glycoengineering, adipose stem cell glycoengineering, stem cell differentiation

## Abstract

This report describes novel thiol-modified *N*-acetylmannosamine (ManNAc) analogs that extend metabolic glycoengineering (MGE) applications of Ac_5_ManNTGc, a non-natural monosaccharide that metabolically installs the thio-glycolyl of sialic acid into human glycoconjugates. We previously found that Ac_5_ManNTGc elicited non-canonical activation of Wnt signaling in human embryoid body derived (hEBD) cells but only in the presence of a high affinity, chemically compatible scaffold. Our new analogs Ac_5_ManNTProp and Ac_5_ManNTBut overcome the requirement for a complementary scaffold by displaying thiol groups on longer, *N*-acyl linker arms, thereby presumably increasing their ability to interact and crosslink with surrounding thiols. These new analogs showed increased potency in human neural stem cells (hNSCs) and human adipose stem cells (hASCs). In the hNSCs, Ac_5_ManNTProp upregulated biochemical endpoints consistent with Wnt signaling in the absence of a thiol-reactive scaffold. In the hASCs, both Ac_5_ManNTProp and Ac_5_ManNTBut suppressed adipogenic differentiation, with Ac_5_ManNTBut providing a more potent response, and they did not interfere with differentiation to a glial lineage (Schwann cells). These results expand the horizon for using MGE in regenerative medicine by providing new tools (Ac_5_ManNTProp and Ac_5_ManNTBut) for manipulating human stem cells.

## 1. Introduction

**Figure 1:**
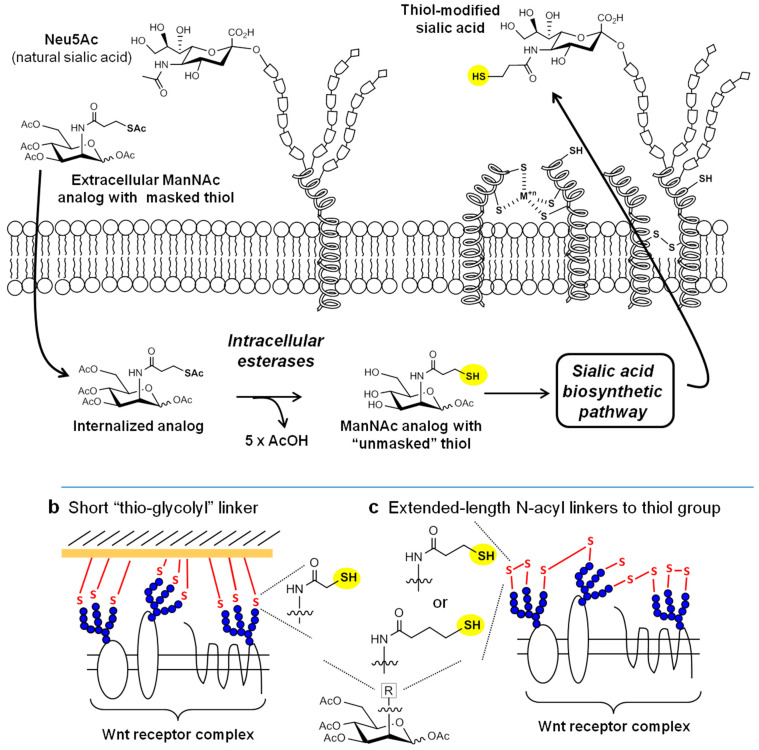
Overview of thiol-based metabolic glycoengineering (MGE). (**a**) Thiol groups can be metabolically installed into cell surface sialic acids by incubating cells with exogenously-supplied ManNAc analogs that have thiol groups masked by acetyl esters [5,8]; these protecting groups facilitate uptake into cells and then are removed (along with the analog’s hydroxyl esters [9,10]) by intracellular esterases [11,12]. The resulting thiol-modified ManNAc analogs intercept the sialic acid biosynthetic pathway and replace naturally-occurring sialic acids (e.g., Neu5Ac, the predominant form of this sugar in humans) in cell surface glycans with their thio-glycosialoside counterparts. (**b**) In previous work, we described the “scaffold-dependent” activity of thiol-modified sialic acids with short linker lengths between the thiol group and core sugar; an example of a ManNAc analog that installs such a sialic acid into cellular glycans is Ac_5_ManNTGc [5] (Figure 2). (**c**) In the current report, we describe thiol-modified analogs (e.g., Ac_5_ManNTProp and Ac_5_ManNTBut, also shown in Figure 2) with longer linkers (i.e., with 3 or 4 carbon atoms in the *N*-acyl linker) between the thiol and core sugar that have enhanced scaffold-independent biological responses.

Metabolic glycoengineering (MGE) is a three decades old “chemical biology’ technology [1,2] that is showing renewed vigor for therapeutic and regenerative applications as new chemically-modified monosaccharide analogs and delivery modalities are developed [3,4]. This report describes thiol-modified ManNAc analogs that replace Neu5Ac, the predominant natural form of sialic acid in humans, with thiol-modified variants of this sugar on the surfaces of human cells [5,6,7] as outlined in Figure 1a.

**Figure 2:**
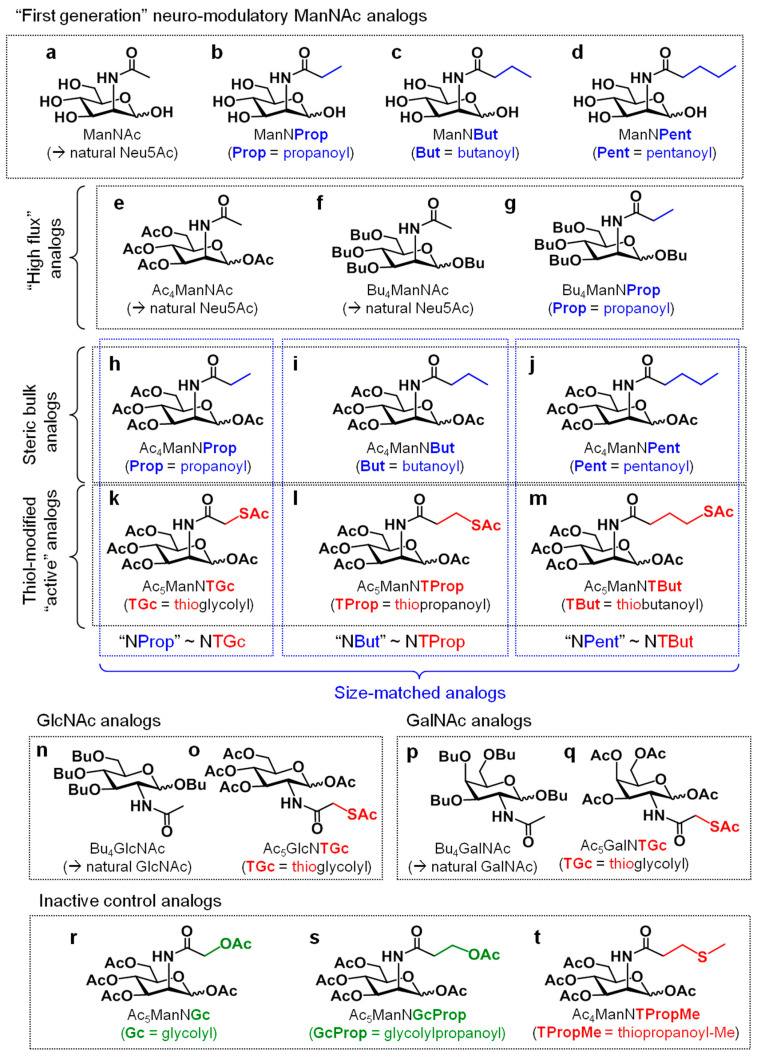
Thiol-modified (and control) analogs (**a**–**t**). Chemical structures of hexosamine analogs mentioned in this report (note that not all analogs shown were used in all experiments and the names used here conform to those used in previous publications, e.g., Prop, But, and Pent refer to the propanoyl-, butanoyl-, and pentanoyl-modified hexosamines [1,22,23] and ‘TGc’ denotes thio-analog counterparts to the glycolyl sugars [5,20]).

The manipulation of embryonic and stem cells illustrates how MGE has biomedical potential by modulating biological responses. Early work, published in a series of papers by Werner Ruetter and colleagues in the 1990s, established that ManNAc analogs such as ManNProp, ManNBut, and ManNPent (Figure 2b–d) altered the fate of neonatal neural cells from rodents [13,14,15,16]. The presumed mechanism for these hexosamine analogs was through metabolic conversion to the corresponding *N*-acyl-modified sialic acids followed by their biosynthetic incorporation into cell surface sialoglycosides in place of natural sialic acids. Once on the cell surface, the non-natural sialic acids (e.g., Sia5Prop, Sia5But, or Sia5Pent) interfere with receptor or cell adhesion interactions. Interestingly, although sialic acids occur ubiquitously on all types of mammalian cells and the incorporation of non-natural sialic acids is likewise widespread across the cell and tissue types [2], observable perturbations of cell biology or physiology are relatively rare upon MGE intervention. In other words, MGE is often considered to be a “silent” technique to label biological molecules with negligible or minor interference with cellular processes. In reality, however, MGE can selectively alter a limited repertoire of outcomes at certain times in an organism’s development (e.g., in early-stage Drosophilia [17] or neonatal rodent brain cells [13]) or in certain disease states (e.g., cancer [3]).

The relatively narrow impact of non-natural sugar analogs on a small subset of cells in an organism provides an opportunity to use MGE to selectively modulate biology, with the pioneering studies by Reutter and colleagues pointing towards neural tissue as an enticing endpoint [13,14,18]. Possibilities for precise control of neural cell biology were evident by the effects of subtle responses—and sometimes more profound differences—elicited by ManNProp and ManNBut, which differ in chemical structure by only one methylene unit in the sugar’s *N*-acyl moiety; for example, ManNBut alters the polysialic acid display on the neural cell adhesion molecule (NCAM) while the analogs with shorter *N*-acyl groups do not [19]. Based on several lines of intriguing evidence that show that increased steric bulk of the *N*-acyl moiety of ManNAc analogs can modulate biochemical responses associated with neural cell fate, we were curious what effect changes in chemical functionality would have. Accordingly, we synthesized ManNAc analogs (Ac_5_ManNGc (Figure 2r), previously reported by the Schnaar group for use in neural regeneration [20], and Ac_5_ManNTGc (Figure 2k), a compound designed by our team [8,21]) that install the glycolyl and thiol-glycolyl forms of sialic acid into cellular glycans, respectively.

Our then-new (in 2006) thiol-modified analog Ac_5_ManNTGc triggered neural cell differentiation in human embryoid body derived (hEBD) stem cells in the absence of the Wnt signaling proteins (or other biochemical stimuli) typically required for this biological response [5]. Noteworthily, Wnt pathway upregulation only occurred when the cells were grown on a gold-covered surface where the thiol-modified cell surface sialic acids could form high-affinity bonds with the substrate (Figure 1b). By contrast, differentiation was not observed in Neu5Gc-expressing cells treated with Ac_5_ManNGc, which lacked sialic acid-displayed thiol groups. The non-natural glycoengineered cell adhesion between the gold surface and the thiol groups of Sia5TGc in Ac_5_ManNTGc-treated hEBD cells increased the abundance of Wnt proteins (e.g., Wnt 10 and 3) required for neural differentiation [5]. We subsequently showed unusual biological responses for Ac_5_ManNTGc-treated Jurkat cells that also were scaffold-dependent [7,24]. In particular, this leukemic T-cell derived line, which normally grows in suspension, shifted to adhesive growth when cultured in the presence of nanofibers coated either with gold or maleimide, which like gold forms high-affinity bonds with sialic acid-presented thiol groups (Figure 1b). Remarkably, these cells secreted copious amounts of extracellular matrix (ECM) components when treated with Ac_5_ManNTGc, which (to our knowledge) is unprecedented for blood cells. Although not fully characterized at a mechanistic level, these experiments with Jurkat cells established a second scenario where thiol-modified cell surface sialic acids combined with a high-affinity growth substrate altered cell fate.

Although the ability of Ac_5_ManNTGc to modulate hEBD and Jurkat cell fates when combined with a complementary scaffold was scientifically interesting, this approach was not amenable for translational research because of difficulties in developing in vivo applications that depend on a gold-plated surface or other appropriately functionalized scaffolds. Accordingly, we consider the novel Ac_5_ManNTProp and Ac_5_ManNTBut analogs described in this report to be transformative for applying thiol analog-based MGE for tissue engineering and regenerative medicine applications.

## 2. Materials and Methods

### 2.1. Cell Culture

Human neural stem cells (hNSCs) derived from human embryonic stem cell line H9 (WiCell, Madison, WI, USA) were seeded at a density of 100,000 cells/well into 24-well culture plates in triplicate [25]. The plates were pre-coated with polyornithine (MilliporeSigma, Burlington, MA, USA, 10 μg/mL, 37 °C for 2.0 h) and laminin (MilliporeSigma, 10 μg/mL, RT overnight) [26]. The cell culture medium consisted of Neurobasal medium supplemented with 2.0 mM L-glutamine, 1xB27 supplement (ThermoFisher, Waltham, MA, USA), 10 ng/mL leukemia inhibitory factor (LIF from Invitrogen, Carlsbad, CA, USA), and 20 ng/mL human recombinant basic fibroblast growth factor (bFGF; MilliporeSigma). Media was replaced every other day. Cell numbers were determined by using a Coulter cell counter as we described previously [27].

Human adipose stem cells (hASCs) were obtained from Warren Grayson’s laboratory (Translational Tissue Engineering Center and Department of Biomedical Engineering, The Johns Hopkins University); these cells were isolated from lipoaspirate tissue of a 39-year-old Caucasian female donor under an institutional review board-approved protocol, as previously described [28]. The hASCs were grown in proliferation medium: high-glucose DMEM with 10% fetal bovine serum (FBS), 1.0% of a 100× stock solution of penicillin/streptomycin (P/S), all from Invitrogen, and 1.0 ng/mL bFGF. Adipogenic differentiation was carried out for hASCs supplemented with adipogenic induction media (1.0 μM dexamethasone, 200 μM indomethacin, 500 μM methylisobutylxanthine, 10 μg/mL insulin, 1.0% of 100× stock solution of penicillin/streptomycin, and 10% FBS in high-glucose DMEM) [29]. Cells were differentiated for seven days and fixed for histological analysis. Schwann cell differentiation from hASCs followed a previously described procedure [30]; briefly, hASC cultures were incubated for 24 h in an expansion medium containing 1.0 mM β-mercaptoethanol (Sigma-Aldrich, St. Louis, MO, USA), and then β-mercaptoethanol was removed and replaced with 35 ng/mL all-trans-retinoic acid (Sigma-Aldrich) for another 3 days. After the pre-condition treatment, a cell differentiation medium containing 5.0 ng/mL platelet-derived growth factor (Gibco, Gaithersburg, MD, USA), 10 ng/mL bFGF, 200 ng/mL NRG1-β1, and 14 µM forskolin (Sigma-Aldrich) was added. The cells were incubated for two weeks under these conditions. The fresh medium was changed every three days.

### 2.2. Treatment of Cells with Sugar Analogs

The novel thiol-modified ManNAc analogs (Ac_5_ManNTProp and Ac_5_ManNTBut) were synthesized and characterized as described in the Supplementary Materials; other analogs were synthesized as previously published [7,8]. All analogs were lyophilized and stored at −80 °C until needed for experiments, at which point 50 mM stock solutions were prepared in ethanol and stored at −20 °C for no longer than six weeks. Analog, or an equal volume of ethanol as the “negative” control, was added to the culture plate prior to cell seeding, and cells were incubated for two days for toxicity and thiol expression studies [7,11]. The resulting cell-surface thiols (CSTs) were detected and quantified by labeling the cells with (+)-biotinyl-3-maleimidopropionamidyl-3,6-dioxaoctanediamine, followed by staining with fluorescein-conjugated avidin and quantification by flow cytometry [11]. For the cell differentiation study, cells were seeded on day 1, and the analogs were added to the proliferation medium of hNSCs and the differentiation medium of hASCs on day 2, and the media were replenished every other day.

### 2.3. Oil Red O Staining

For adipogenic differentiation, hASCs in adipogenic and control media were fixed with 4.0% paraformaldehyde at day 7 to assess intracellular lipid vesicles using Oil red O staining [31]. Briefly, 60% isopropanol (Sigma-Aldrich) was added to the wells and incubated for 5 min at RT. After removing the isopropanol, cells were stained with 0.3% Oil red O (Sigma-Aldrich) for 15 min. After washing, hematoxylin was added for 1.0 min to counterstain the cells before imaging. The stain was then extracted using 99% isopropanol and quantified using the microplate reader (Molecular Devices, San Jose, CA, USA) at 530 nm absorbance.

### 2.4. Immunofluorescence Staining

Cells were fixed with 4.0% paraformaldehyde, permeabilized with 0.1% Triton X-100, and blocked with 2.5% BSA buffer. Primary antibody against β-tubulin III (TUJ1, MilliporeSigma) and Microtubule-associated protein 2 (MAP2, MilliporeSigma) for hNSCs and S-100 (MilliporeSigma) for hASCs were applied in blocking buffer overnight at 4 °C. Cells were then washed in PBS and treated with secondary antibodies coupled to FITC (Jackson Immuno Research, West Grove, PA, USA) for 60 min. After two washes in PBS, cells were stained with 4′,6′-diamidino-2-phenylindole (DAPI, Invitrogen). In control sections, primary antibodies were omitted from the staining procedure. Microscopy was performed using a Nikon TE2000 fluorescence microscope. Three samples from each condition were examined under the microscope.

### 2.5. Reverse Transcriptase Polymerase Chain Reaction Analysis

RNA was isolated by using Trizol (Invitrogen) following standard protocols. RNA concentrations were obtained using a Nanodrop 2000 spectrophotometer. One microgram of RNA was reverse-transcribed to cDNA using the High Capacity RNA-to-cDNA Kit (Applied Biosystems, Foster City, CA, USA). Quantitative reverse transcriptase-polymerase chain reaction (qRT-PCR) was performed on the cDNA using a Step One Plus system (Applied Biosystems) and TaqMan primers and reagents (Applied Biosystems). Relative expression levels of each gene compared to GAPDH were determined using the 2^−ΔΔ*Ct*^ method. The reference condition used for the comparison of cells was cells incubated under comparable conditions with the solvent vehicle; all data were normalized to this condition.

### 2.6. Statistical Analysis

Experiments were performed in triplicate and repeated at least three times. All data presented in the study were mean ± standard deviation unless otherwise noted. Between-group comparisons were tested with t-test or one-way ANOVA. A *p*-value less than 0.05 was considered statistically significant.

## 3. Results

### 3.1. Biological Responses of hNSCs Treated with Control and Thiol-Modified ManNAc Analogs

**Figure 3:**
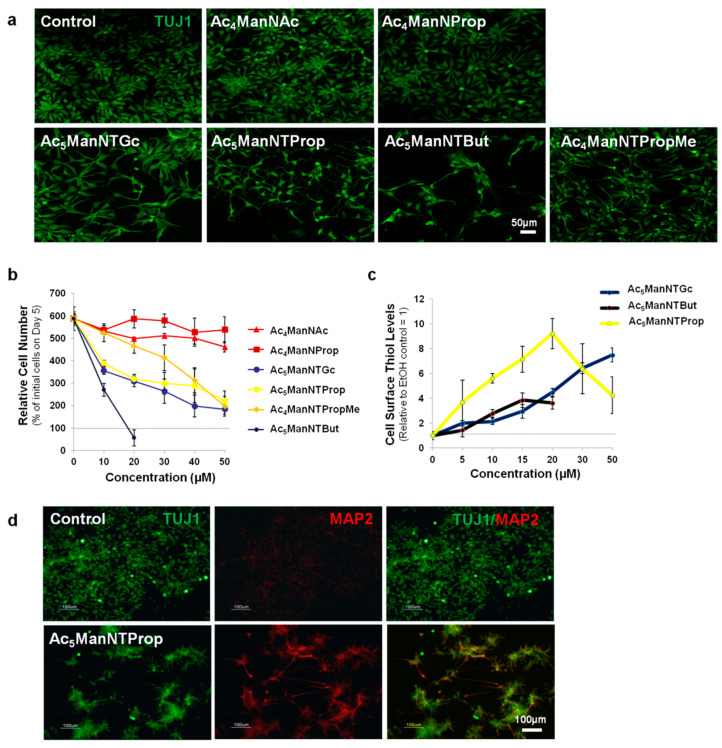
Biological responses of human neural stem cells (hNSCs) treated with thiol-modified ManNAc analogs. (**a**) Fluorescence microscopy images (stained by TUJ1, an antibody for class III β-tubulin protein) of control and Ac_4_ManNAc- and Ac_4_ManNProp-treated hNSCs showed that these unthiolated ManNAc analogs did not noticeably alter cell morphology (top row). Comparison of these results with the three thiol-modified analogs showed clear morphological changes and reduced cell numbers upon Ac_5_ManNTProp and Ac_5_ManNTBut treatment, with weaker responses observed for Ac_5_ManNTGc (bottom row). In addition, the thio-ether analog Ac_4_ManNTPropMe is shown in the bottom row; despite containing a sulfur atom, this analog cannot be metabolically converted to a thiol and elicits biological responses similar to those of the non-thiolated analogs shown in the top row. (**b**) Dose-response experiments showed that only Ac_5_ManNTBut was cytotoxic at concentrations up to 50 μM after Day 5 (i.e., there were fewer cells on Day 5 than were plated on Day 0); the other analogs slowed the growth of the cells but did not induce cell death, consistent with the cell densities shown in (**a**). (**c**) Cell surface thiols were quantified by flow cytometry-based on our published protocols [21] with ethanol control samples arbitrarily set to a value of 1.0. (**d**) Evaluation of neuronal differentiation by TUJ1 (green) and MAP2 (red, a marker of mature neurons) in ethanol control (top row) and Ac_5_ManNTProp-treated (bottom row) hNSCs. In these experiments, hNSCs were plated on laminin-coated surfaces on Day 0, analogs were added at 20 μM concentration on Day 1 and replenished every second day. The cells were stained and imaged on Day 5 and Day 9 for (**a**,**d**), respectively. Experiments were performed in triplicate and repeated three times. The cells in (**a**) are shown at higher magnification to depict morphology more clearly. The scale bar was 50 and 100 µm for (**a**,**d**), respectively.

In our first set of experiments, we treated hNSCs with 20 µM of several ManNAc analogs (Figure 3). Cell morphology and proliferation in samples treated with solvent vehicle or non-thiol modified analogs (Figure 3a, top row) were not noticeably different than that of control cells. The control cells were treated with ethanol, the solvent vehicle for the ManNAc analogs. In all cases, less than 0.1% (*v*/*v*) of ethanol was added to the cell cultures; in our numerous, previous MGE experiments in multiple cell types, we have not observed any solvent effects at these levels of ethanol [32,33,34,35,36,37]. Upon testing the thiol-modified ManNAc analogs, Ac_5_ManNTGc showed indications of altered morphology and lower cell number whereas the newly developed analogs (Ac_5_ManNTProp and Ac_5_ManTBut) showed clear morphological differences in hNSCs after incubation for five days (Figure 3a, lower row); in addition, cell number progressively decreased with longer *N*-acyl chain length for the thio-analogs. A statistically significant dose-dependent reduction in cell number after 5 days of incubation compared to the control cells was confirmed by cell counts (Figure 3b). The dose-response curves (along with additional routine toxicity assays such as live/dead staining and annexin V flow cytometry assays, not shown) indicated that Ac_5_ManNTProp and other analogs tested were not cytotoxic to hNSCs at concentrations up to 50 µM, with one exception. The outlier, Ac_5_ManNTBut, was cytotoxic at concentrations at >10 µM, as indicated by the presence of fewer cells after five days than were plated on Day 0.

We next tested the cell surface incorporation of thiol-modified sialic acids in Ac_5_ManNTGc-, Ac_5_ManNTProp-, and Ac_5_ManNTBut-treated hNSCs (Figure 3c). The maximal response to Ac_5_ManNTProp occurred at ~20 µM, where an approximately 9-fold increase in thiol expression was seen compared to that of the ethanol-treated control. For the Ac_5_ManNTGc-treated cell surfaces, the display of thiol groups increased approximately linearly up to a concentration of 50 µM but only reached ~7-fold higher levels than that of control cells. In Ac_5_ManNTBu-treated cells, the toxicity of this analog at concentrations greater than 20 µM prevented analysis of surface thiol display at higher concentrations.

Because of the potential cytotoxicity of Ac_5_ManNTBut to hNSCs, our next experiment—designed to further explore the biological consequences of the display of thiolated sialic acids on these cells—focused on Ac_5_ManNProp, which induced morphological changes in the absence of cell death in our earlier experiments (Figure 3a). We showed the prolonged incubation of hNSCs (for 9 days) with 20 µM Ac_5_ManNTProp resulted in the appearance of βIII-tubulin-positive cells that co-expressed MAP2 (Figure 3d), indicating that this thiol-modified analog promoted the generation of neuronal subtypes not seen in the ethanol, Ac_4_ManNAc, Ac_4_ManNProp, or Ac_5_ManNTGc-treated control cells. In addition, the thio-ether analog (Ac_4_ManNTPropMe), which does not install free thiol groups on the cell surface, did not induce clear indications of neuronal differentiation; instead, cell morphology and counts were similar to that of non-thiol controls (Figure 3a,b).

### 3.2. Impact of Thiol-Modified ManNAc Analogs on Wnt Signaling in hNSCs

**Figure 4:**
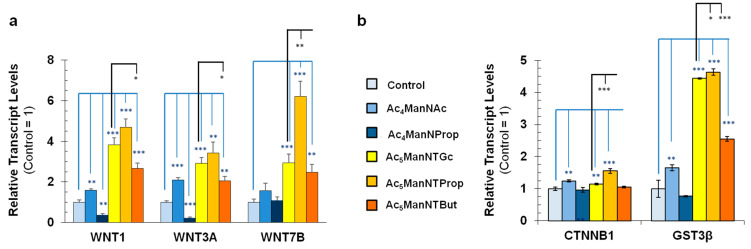
Wnt pathway activation by thiol-modified ManNAc analogs in hNSCs. (**a**) qRT-PCR results showed WNT1, WNT3a, and WNT7b were statistically upregulated more strongly by thiol-modified ManNAc analogs than by sized-matched non-thiolated analogs when compared to the untreated control as indicated by the *p* values annotated using the blue lines; as shown by the black lines, statistically significant results were also observed for the newly-developed Ac_5_ManNTProp and Ac_5_ManNTBut thio-analogs compared to Ac_5_ManNTGc, our previous thiolated ManNAc analog. (**b**) Transcript analysis of CTNNB1 (β-catenin) and particularly for GSK3β showed stronger upregulation by thiol-modified ManNAc analogs compared to their size-matched controls; in addition, statistically significant differences were again observed between the thio-analogs with both β-catenin and GSK3β most strongly upregulated in Ac_5_ManNTProp-treated hNSCs. (* indicates *p* < 0.05, ** indicates *p* < 0.01, *** indicates *p* < 0.001; if no value is shown, the results were not significantly different).

In our previous studies, we demonstrated that Ac_5_ManNTGc, our short linker chain thiol-modified ManNAc analog, upregulated Wnt signaling in hEBD cells, but only when the cells were grown on gold surfaces [5]. These prior results are consistent with the present study where hNSCs grown without a high-affinity growth substrate for thiols elicited weak, if any, responses when grown with Ac_5_ManNTGc (Figure 3). Here, based on the stronger morphological and cell number responses observed for Ac_5_ManNTProp and Ac_5_ManNTBut in the absence of a complementary growth surface for thiols, we tested whether biochemical endpoints linked to Wnt signaling could be upregulated in hNSCs under similar conditions using these new extended linker length, thiol-modified analogs. Upregulation of Wnt-associated genes was shown by qRT-PCR analysis of WNT1, WNT3a, and WNT7b in hNSCs treated with a panel of control and thio-analogs with the quantitatively largest results observed in Ac_5_ManNTProp-treated cells (Figure 4a). In addition, β-catenin (CTNNB1), an indicator of Wnt pathway activation, and glycogen synthase kinase 3β (GSK3β), a multifunctional serine/threonine kinase in the Wnt pathway abundant in the developing central nervous system were upregulated by several analogs, with the strongest responses again observed for Ac_5_ManNTProp-treated cells (Figure 4b).

### 3.3. Thiol-Modified ManNAc Analogs Suppress Adipocyte Differentiation in hASCs

**Figure 5:**
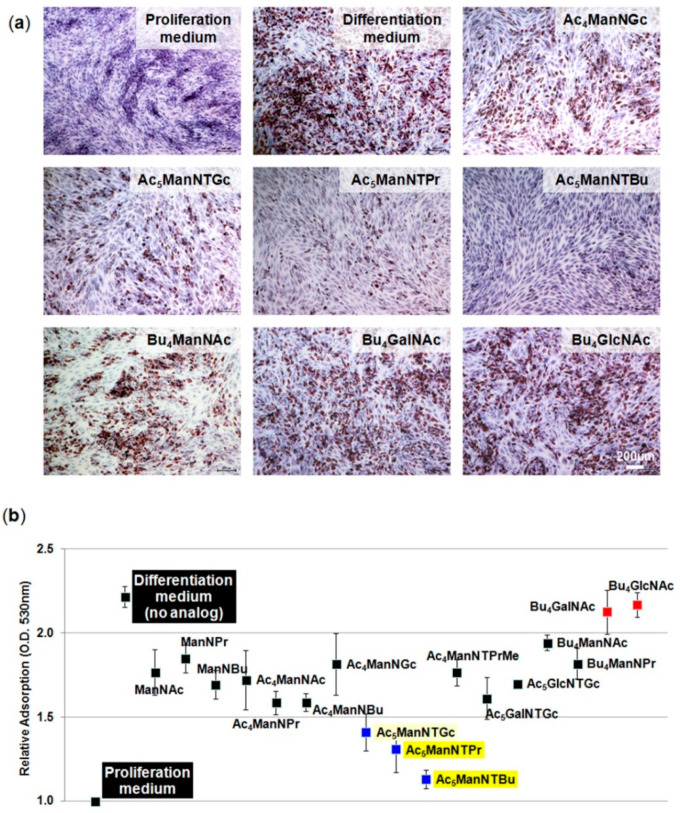
Oil Red O staining of human adipose stem cells (hASCs). Oil red O staining of hASCs exposed to selected analogs at 50 μM (except 1.0 mM for ManNAc, ManNProp, and ManNBut) for 7 days in an adipogenic medium. The dye retained by the lipid vacuoles is illustrated for selected analogs in (**a**) and was measured quantitatively by optical density (O.D.) values at 530 nm in panel (**b**). Except for the picture labeled “Proliferation medium,” all cells were grown in differentiation medium and the picture labeled “Differentiation medium” is the solvent control (i.e., an equal volume of ethanol was added as-used to deliver hexosamine analogs to the other analog-treated samples). Spectrophotometric analysis data were normalized to cell number and the adsorption of cells grown in proliferation media, which was set at a value of 1.0.

One goal of this study was to investigate whether thiol-modified analogs used in MGE constitute a broad-based tool to modulate stem cell biology. Therefore, based on the ability of thiol-modified ManNAc analogs to modulate biology in two cell lines that were predisposed towards neural cell fates by upregulating Wnt signaling in hEBD cells in previous studies [5] with similar indications of Wnt activation in hNSCs in the current study, we were intrigued to test a third and unrelated type of stem cell. We selected human adipose stem cells (hASCs) that, although capable of neuronal (and other types of) differentiation, primarily differentiate into mature adipocytes in situ. Interestingly, in hASCs, Wnt signaling suppresses the primary differentiation fate of these cells, which is adipocyte formation [38,39]. Accordingly, based on the precedent that thiol-modified analogs upregulate Wnt-associated endpoints across cell lines, we tested adipogenesis upon treatment of primary hASCs with thiol-modified and control analogs (Figure 5).

In these experiments, cells cultured in a proliferation medium served as a baseline, where negligible differentiation into mature adipocytes occurred (Figure 5a, top row, leftmost panel). As a positive control for adipogenesis, cells were incubated in a differentiation medium, which resulted in ~60% of cells differentiating into adipocytes as indicated by Oil red O staining of lipid droplets in the cells (Figure 5a, top row, middle panel). The addition of ManNAc analogs at 20 µM suppressed adipocyte differentiation to different extents (depending on the analog) when measured by Oil red O quantification (Figure 5b). This suppression was not observed for GalNAc or GlcNAc analogs (i.e., Bu_4_GalNAc or Bu_4_GlcNAc), which are the other two mammalian hexosamines in addition to ManNAc. Because neither GalNAc or GlcNAc nor their thio-glycolyl counterparts Ac_5_GalNTGc and Ac_5_GlcNTGc directly modulated sialylation, this result suggested that sialic acid biosynthesis, which is the primary metabolic fate of ManNAc, is involved in adipocyte differentiation. Indirect effects on sialylation through the conversion of GlcNAc to ManNAc [40] were discounted by increasing GlcNAc levels through Bu_4_GlcNAc treatment. Of particular interest was the response to the three thiol-modified ManNAc analogs (Ac_5_ManNTGc, Ac_5_ManNTProp, and Ac_5_ManNTBut, highlighted in yellow in Figure 5b), where suppression of adipocyte differentiation increased with linker length, rendering Ac_5_ManNTBut the most potent analog.

**Figure 6:**
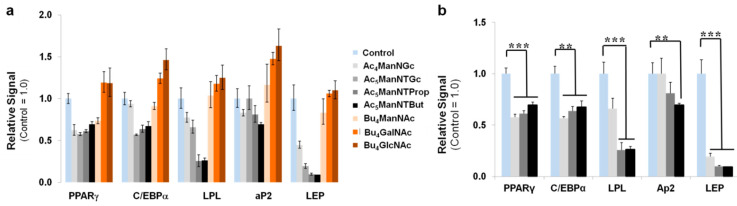
Transcript analysis of adipogenic differentiation in hASCs. (**a**) qRT-PCR analysis for adipogenic differentiation markers after Day 7 of treatment with a panel of analogs in adipogenic medium. Markers tested are PPARγ (peroxisome proliferator-activated receptor γ), C/EBPα (CCAAT/enhancer-binding protein α), LPL (lipoprotein lipase, an early indicator of adipocyte differentiation), aP2 (fatty acid-binding protein 4, FABP4, intermediate marker of adipocyte differentiation), and LEP (leptin, a late marker of adipocyte differentiation). Statistical analysis of the RT-PCR was carried out using the (2^−ΔΔ*Ct*^) method, which calculates the relative changes in mRNA levels normalized to an endogenous reference (GAPDH) relative to a calibrator (without analog treatment) that serves as the control group and was expressed as fold change; these analyses showed the strongest suppression occurred in Ac_5_ManNTProp- and Ac_5_ManNTBut-treated hASCs (**b**). (** indicates *p* < 0.01 and *** indicates *p* < 0.001).

To quantify gene expression patterns associated with adipogenesis, we conducted the qRT-PCR analysis for adipogenic differentiation markers after one week of treatment of hASCs with several of the test analogs in the adipogenic medium. Markers tested were PPARγ (peroxisome proliferator-activated receptor γ), C/EBPα (CCAAT/enhancer-binding protein α), LPL (lipoprotein lipase, an early marker of adipocyte differentiation), aP2 (fatty acid-binding protein 4, FABP4, intermediate marker of adipocyte differentiation), and LEP (leptin, a late marker of adipocyte differentiation). In these experiments, Bu_4_GlcNAc and Bu_4_GalNAc, which increase hexosamine biosynthesis, failed to suppress the expression of these adipogenic genes (Figure 6a) and indeed, showed a trend towards increased expression, which is consistent with the Oil red O staining results shown in Figure 5a, where these two compounds appeared to increase lipogenic differentiation. By contrast, the ManNAc analogs were generally suppressive with Ac_5_ManNTProp and Ac_5_ManNTBut, the two analogs with thiol groups on extended *N*-acyl linker arms, showing the strongest and highly statistically significant suppression of markers for adipogenic differentiation, especially for LPL and LEP (Figure 6b).

### 3.4. Glial Cell Differentiation of hASCs

**Figure 7:**
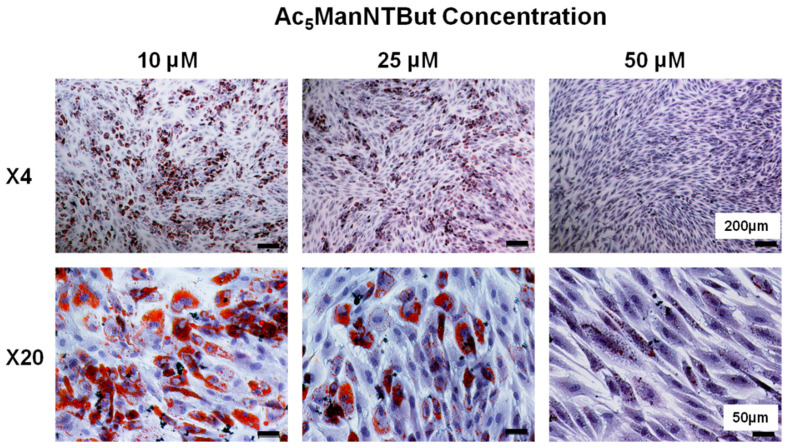
Dose-response of Ac_5_ManNTBut-treated hASCs. hASCs were cultured in adipogenic differentiation medium with the indicated concentrations of Ac_5_ManNTBut and visualized by phase-contrast microscopy after Oil red O staining on Day 7 at 4× or 20× magnification. Lipid droplets were stained red and nuclei were stained blue.

An important observation from screening the panel of analogs at 20 µM (Figure 5 and Figure 6) was that unlike for hNSCs where Ac_5_ManNTBut was mildly toxic, this analog showed no cytotoxicity towards hASCs. A dose-response to evaluate suppression of adipogenesis was then conducted for Ac_5_ManNTBut, which showed progressive loss of Oil red O staining at 10, 25, and 50 µM, again without loss of cell viability (Figure 7). The mechanism by which hASCs resist the potential cytotoxicity of Ac_5_ManNTBut observed in hNSCs (Figure 3b) is unknown, but the observation that complete inhibition of adipogenic differentiation was possible in the absence of adverse effects on viability or proliferation discounts the possibility that this outcome resulted from analog toxicity. This finding was important because it, in theory, opens the door to using Ac_5_ManNTBut to suppress the primary differentiation fate of hASCs, thereby improving the yield and homogeneity of alternative outcomes; for example, the use of hASCs for neural differentiation.

**Figure 8:**
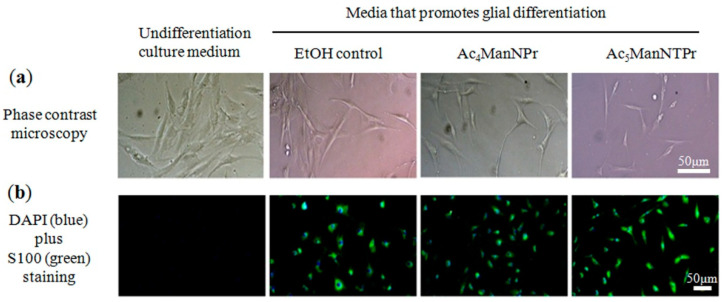
Schwann cell (SC) differentiation of hASC. (**a**) Morphological and physiological changes following ASC glial differentiation. hASCs showed a flattened fibroblast-like morphology that adopted a spindle elongated, SC-like morphology after incubation in differentiation medium. (**b**) Immunofluorescence staining indicated differentiated hASCs expressed the S100 protein, a Schwann cell marker. Scale bars = 50 µm.

Accordingly, we next tested whether these compounds generally suppressed hASC differentiation or whether this effect was confined to adipogenic fates. Keeping our focus on neural cell fates, we evaluated glial differentiation by incubating hASCs in the appropriate differentiation medium that does not promote glial differentiation. As shown, morphological and physiological changes consistent with glial differentiation were observed only with the differentiation medium (Figure 8a); specifically, hASCs showed a flattened fibroblast-like morphology that adopted a spindle elongated, Schwann cell-like morphology after incubation in differentiation medium. Moreover, immunofluorescence staining showed that cells incubated in differentiation media expressed the S100 protein, a Schwann cell marker (Figure 8b). The addition of Ac_4_ManNProp and Ac_5_ManNTProp to the media did not prevent morphological changes to the cells or ablate S100 expression. These results support the notion that thiol-modified ManNAc analogs are not detrimental to the induction of non-adipogenic fates for hASCs while suppressing adipogenesis.

## 4. Discussion

This report builds on previous evidence that ManNAc-based MGE can influence the fate of embryonic cells. This line of work dates from the late 1990s when Reutter’s group showed that increased steric bulk at the *N*-acyl position of ManNAc analogs, which is reflected in the corresponding *N*-acyl moieties of non-natural cell surface sialic acids, modulated the fate of embryonic cells [13,14,15,16]. Our team later extended this line of work with thiol-modified ManNAc analogs, most notably the “thio-glycolyl” *N*-acyl modification found in Ac_5_ManNTGc [8,21] (Figure 2k) in hEBD [5] and Jurkat [7,24] cells. In the follow-up studies described in the current report, we developed new ManNAc analogs that have “stand-alone” biomodulatory activities derived from changes to their chemical structures and described examples of these activities in two types of human stem cells (neural (hNSCs) and adipose (hASCs) stem cells).

To briefly recap analog design considerations, the field of MGE has been dominated by chemical biologists who have long pursued the goal of introducing bio-orthogonal chemical functional groups into cellular glycans using hexosamine analogs. These efforts were pioneered by the Bertozzi group who demonstrated almost 25 years ago the concept of introducing non-natural chemical functional groups into sialosides with ketones [41,42] and azides [43]. Since then, dozens of chemical functional groups have been introduced into MGE analogs (as reviewed by us [2,3,4,44,45] and others [14,18,46,47,48]). Almost always, chemical functional groups used in MGE analogs have been designed to achieve bio-orthogonality to enable chemoselective ligation in the physiological milieu [43]. In other words, these compounds are specifically designed to not react with all chemical functional groups normally present in living systems. It should be noted that some functional groups, like the Reutter group’s original set of *N*-acyl elongated analogs (Figure 2b–d), can elicit biological responses through steric effects, but as noted above, these responses are less robust than those of our novel thiol-modified analogs.

The thiol group is fundamentally different than existing analogs because it has “stand-alone” chemical reactivity in a biological system without any need for outside intervention because it does not require a second, complementary functional group (for example, an alkyne for bio-orthogonal ligation to an MGE-expressed azide) or irradiation to activate photoactivated functional groups [49]. By contrast, a non-naturally installed thiol group can interact with naturally-occurring, endogenous thiols found in a cell’s nano- and microenvironment or through cis interactions with surrounding biomacromolecules on the same cell. Moreover, the reactivity of thiol groups can be modulated by the redox potential of the modified glycan’s surroundings. For example, the inside of a cell is a reducing environment, ensuring that the thiol group remains in the reduced (“free”) form inside a cell. The extracellular milieu, by contrast, is oxidizing, resulting in 90–95% of MGE-installed thiol-modified sialic acids being in the oxidized (cross-linked) form in ordinary cell culture conditions [5,21]. This feature provides a reservoir of thiol groups in one state of activity that can be rapidly shifted to another state by mild tris(2-carboxyethyl)phosphine (TCEP) reduction, thus increasing the abundance of thiol groups in the free, unreduced form. Depending on the endpoint, such reduction can either increase or decrease thiol-mediated activity. For example, if the goal is to label surface thiols with imaging agents [5,21] (or drugs [50]), mild reduction increases activity. By contrast, if the activity being sought relies on disulfide crosslinking (e.g., as shown in Figure 1c), the mild reduction will decrease activity.

In the current study, we found that by increasing the linker length of the *N*-acyl group of thiol-modified ManNAc analogs (e.g., from two carbons in Ac_5_ManNTGc (Figure 2k), to three carbons in Ac_5_ManNTProp (Figure 2l), and to four carbons in Ac_5_ManNTBut (Figure 2m)), the potency of each analog in eliciting biomodulation increased correspondingly. Importantly, the three- or four-carbon linkers (in Ac_5_ManNTProp and Ac_5_ManNTBut, respectively), elicited strong responses in the absence of a complementary high-affinity scaffold, which was required in previous studies for Ac_5_ManNTGc-mediated activation of neural differentiation in hEBD cells and ECM production in Jurkat cells. At the same time, size-matched non-thiolated analogs (i.e., Ac_4_ManNBut for Ac_5_ManNTProp) showed weaker or negligible responses. A pitfall, however, for using Ac_5_ManNTBut for hNSC glycoengineering was that this analog exhibited strong growth inhibition and modest cytotoxicity at concentrations required for modulation of hNSCs. By contrast, Ac_5_ManNTProp provided beneficial responses in hNSCs at non-cytotoxic doses.

Indications of Wnt pathway activation observed in Ac_5_ManNTProp-treated hNSCs provide an opportunity for modulating nerve tissue because Wnt signaling cascades are important for the formation of neuronal circuits by controlling neuronal differentiation, axon outgrowth and guidance, dendrite development, synaptic function, and neuronal plasticity [51]. If the ability of this analog to regulate Wnt signaling in hNSCs is rigorously verified in further studies, its safety and scaffold-independent responses position this analog as an enticing molecular tool for use in regenerative medicine and translational hNSC-based therapies. In the current study, further evidence for Wnt pathway activation was gained by evaluating thio-analog treated hASCs; in this case, the opposite effect was expected based on evidence that Wnt signaling suppresses adipogenesis [39,52,53]. Specifically, we experimentally evaluated an expanded panel of hexosamine analogs in hASCs, a type of cells not previously tested with thiol-modified MGE reagents. Our prediction was that if Wnt signaling were upregulated by thio-analogs in these cells, adipogenic differentiation would be suppressed. We found this to be the case (Figure 5, Figure 6 and Figure 7), with stepwise stronger suppression as the *N*-acyl linker length increased from Ac_5_ManNTGc to Ac_5_ManNTProp and then to Ac_5_ManNTBut (Figure 5b). Moreover, Ac_5_ManNTBut was not growth inhibitory or cytotoxic at concentrations that completely suppressed adipogenesis in hASCs. As a final experiment, we showed that Ac_5_ManNTProp suppressed adipogenic differentiation in hASCs but did not hinder glial differentiation (e.g., Schwann cell-like differentiation, Figure 8).

As a final discussion point, we note that we focused on Wnt pathway activation in this report based on our previous studies that linked thiol-modified ManNAc analogs to this pathway [5]. As a caveat, modifying cell-surface glycans via MGE almost certainly modulates additional signaling pathways. For example, in a previous study using non-thiolated analogs, microarray profiling identified 14 pathways modulated by MGE [33], including Wnt signaling, focal adhesion, tight junctions, gap junctions, adipocytokine signaling, and axon guidance, that are of potential relevance to the current results. The diversity of pathways that can be modulated by MGE in general, and in specific ways by subsets of analogs such as the thio-compounds described herein, open the door to pleitropic modulation of cellular physiology in many ways.

## 5. Conclusions

In this report, we describe the enhanced ability of two newly-reported thiol-modified ManNAc analogs (Ac_5_ManNTProp and Ac_5_ManNTBut) to modulate biological responses in human stem cells. Importantly, these responses—which include neural differentiation in hNSCs and suppression of adipogenic differentiation in hASCs—can be achieved in the absence of a chemically compatible growth substrate, which facilitates future in vivo applications and potential clinical translation of these MGE analogs.

## Data Availability

Data available in a publicly accessible repository.

## Supplementary Materials

The following are available online at https://www.mdpi.com/2073-4409/10/2/377/s1, Synthesis, and characterization of Ac_5_ManNTProp and Ac_5_ManNTBut.
