# Supplementary material for: Glycoengineering Human Neural and Adipose Stem Cells with Novel Thiol-Modified N-Acetylmannosamine (ManNAc) Analogs
Source: Cells. 2021 Feb 12;10(2):377. doi: 10.3390/cells10020377 (PMC7918483; doi:10.3390/cells10020377)
Supplement: Supplementary file 1 [file cells-10-00377-s001.pdf]

Supplementary Materials for:

Glycoengineering Human Neural and Adipose Stem Cells with Novel Thiol-Modified N-Acetylmannosamine (ManNAc) Analogs

Jian Du[1], Christian Agatemor[2,3], Christopher T. Saeui[2,3], Rahul Bhattacharya[2,3], Xiaofeng Jia[1,2,4-6,*], and Kevin J. Yarema[2,3,7,8*]

1 Department of Neurosurgery, University of Maryland School of Medicine, Baltimore, MD 21201

2 Department of Biomedical Engineering, The Johns Hopkins School of Medicine, Baltimore, MD, 21205

3 Translational Cell and Tissue Engineering Center, The Johns Hopkins School of Medicine, Baltimore, MD, 21231

4 Department of Orthopedics, University of Maryland School of Medicine, Baltimore, MD 21201

5 Department of Anatomy and Neurobiology, University of Maryland School of Medicine, Baltimore, MD 21201

6 Department of Anesthesiology and Critical Care Medicine, The Johns Hopkins School of Medicine, Baltimore, MD, 21205

7 Department of Chemical and Biomolecular Engineering, The Johns Hopkins University, Baltimore, MD 21218

8 The Sidney Kimmel Comprehensive Cancer Center, Department of Oncology, The Johns Hopkins School of Medicine, Baltimore, MD, 21231.

* Correspondence: X.J., xjia@som.umaryland.edu; K.Y., kyarema1@jhu.edu

Citation: Du, J.; Agatemor, C.; Saeui, C.T.; Bhattacharya, R.; Jia, X.; Yarema, K.J. Glycoengineering Human Neural and Adipose Stem Cells with Novel Thiol-Modified *N*-Acetylmannosamine (ManNAc) Analogs. *Cells* **2021**, *10*, x. https://doi.org/10.3390/xxxxx

Academic Editor: Sebastian P. Galuska; Rüdiger Horstkorte
Received: 6 January 2021
Accepted: 7 February 2021
Published: 12 February 2021

Publisher's Note: MDPI stays neutral with regard to jurisdictional claims in published maps and institutional affiliations.

Copyright: © 2021 by the authors. Licensee MDPI, Basel, Switzerland. This article is an open access article distributed under the terms and conditions of the Creative Commons Attribution (CC BY) license (http://creativecommons.org/licenses/by/4.0/).

Synthesis and characterization of Ac_5ManNTProp and Ac_5ManNTBut

Materials: All solvents and reagents were obtained from Millipore Sigma and they were ACS grade. The following chemicals: bromopropionic acid (97%), potassium thioacetate (98%), oxalic acid dihydrate (>99%), acetic anhydride (>99%), sodium acetate (>99%), and 2-hydroxynaphthaldehyde (technical grade), were also obtained from Millipore Sigma.

Synthesis of 3-(acetylsulfanyl) propanoic acid (2) (Scheme 1a): The compound was synthesized following our previously reported protocol [1]. Briefly, to a 1-L round-bottom flask (RBF) was added bromopropionic acid (**1**) (11.2 g, 73 mmol), acetone (400 mL), and the resulting mixture stirred at room temperature. Potassium thioacetate (12.5, 110 mmol) was added to the reaction mixture, refluxed at 60–70 °C for 30 min, then cooled to room temperature. The mixture was filtered through Celite, washed with 30 mL of acetone (two times), and concentrated in a rotary evaporator. The residue was dissolved in diethyl ether, washed, first, with 200 mL of ice-cold dilute hydrochloric acid (5% v/v), then, with 200 mL of water, dried with anhydrous sodium sulfate and filtered to recover the organic extract. The filtrate was concentrated in a rotary evaporator and purified using vacuum distillation. The product, 3-(acetylsulfanyl) propanoic acid, distilled as the middle fraction at 145 °C at 6–10 mm Hg. Yield = 52%.

Synthesis of 1,3,4,6-tetra-*O*-acetyl-2-ammonium-2-deoxy-*α*-d-mannopyranose oxalate (6) (Scheme 1b). We used our previously reported protocol[1] to synthesize **6**. Briefly, to a 500-mL RBF was added D-mannosamine hydrochloride (**3**) (3 g, 14 mmol), water (3.0 mL), and the mixture stirred until **3** dissolved. Sodium acetate (1.5 g, 18 mmol) was added to the solution followed by 2-hydroxynaphthaldehyde (4.5 g, 26 mmol) and 225 mL of methanol, and the resulting mixture stirred overnight. The mixture was cooled in an ice-water bath for 30 min. The precipitate was filtered using a sintered funnel, washed sequentially with 30 mL of cold water (three times), 30 mL of dichloromethane (two times), and 30 mL of diethyl ether (two times), then dried under vacuum to give **4**.

The precipitate (**4**) (3.7 g, 11 mmol) was weighed into a dry RBF, dissolved in pyridine (85 mL), and the solution cooled with an ice-water bath. Acetic anhydride (85 mL) and 4-dimethylaminopyridine (10 mg) were added to the cooled solution, and after that, the solution was allowed to warm up to room temperature and stirred overnight. The solution was concentrated in a rotary evaporator, and residue resuspended in 50 mL of toluene and reconcentrated (three times) to removed water. The residue was dissolved in 300 mL of dichloromethane, washed with 200 mL of 1.0% (w/v) sodium bicarbonate solution (three times), the organic layer separated and dried using anhydrous sodium sulfate. The organic layer was concentrated and purified using silica gel chromatography as described in our published protocol[1] to give **5**.

The purified residue (**5**) (5.1 g, 9.4 mmol) was added to an RBF, dissolved in 175 mL of acetone, and cooled in an ice-water bath. Oxalic acid dihydrate (5.3 g, 42 mmol) was added to the reaction mixture followed by 75 mL of acetone and stirred for 45 min while cooling in the ice-water bath. After that, the mixture was stirred at room temperature for 60 min, cooled again in an ice-water bath for 15 min, and filtered to collect the precipitate. The precipitate was washed with 60 mL of anhydrous diethyl ether (three times) and dried under vacuum to give **6**.

Synthesis of Ac_5ManNTProp (Scheme 1c): The precipitate (**6**) (1.0 g, 2.3 mmol) and 1-ethyl-3-(3-dimethylaminopropyl) carbodiimide (0.9 g, 4.6 mmol) were added to an RBF. Anhydrous dimethylformamide (DMF) (20 mL) was added and the flask stoppered with a rubber septum. 3-(acetylsulfanyl) propanoic acid (2) (0.7 g, 4.7 mmol) was dissolved in DMF and added to the reaction flask. After 5 min, the reaction mixture was cooled in an ice-water bath, and triethylamine (1.3 mL) was added dropwise with a syringe, then allowed to warm to room temperature and stirred overnight. The reaction was worked up and purified (>95% purity), as described in our previous protocol [1]to give Ac_5ManNTProp. ^{1}H NMR ($CDCl_3$, 400 MHz), Figure S1: δ 6.08 (1 H, d, C*H*), 5.34 (1 H, dd, C*H*), 5.32 (1 H, s, N*H*), 5.21 (1 H, dd, C*H*), 4.68 (1 H, dd, C*H*) 4.30 (1 H, dd, C*H*), 4.07 (2 H, m, CH_2), 3.16 (2 H, m CH_2), 2.60 (2 H, t, CH_2), 2.38 (3 H, s, CH_3), 2.20 (3 H, s, CH_3), 2.12 (3 H, s, CH_3), 2.08 (3 H, s, CH_3), 2.03 (3 H, s, CH_3). ^{13}C NMR ($CDCl_3$, 125 MHz),

Figure S2: δ 196.2, 170.9, 170.6, 170.03, 169.6, 168.2, 91.7, 70.2, 68.9, 65.5, 62.1, 49.3, 36.3, 30.6, 24.8, 20.9, 20.8 (2C), 20.7.

Synthesis of Ac_5ManNTBut (Scheme 1c): Ac_5ManNTBut was prepared using the above protocol except that bromobutyric acid was used in place of bromopropionic acid to prepare **2** (see **Synthesis of 3-(acetylsulfanyl) propanoic acid (2))**. The product was purified[1] and the purity was more than 95%. Ac_5ManNTBut: ^{1}H NMR ($CDCl_3$, 400 MHz), Figure S3: δ 6.19 (1 H, d, C*H*), 6.08 (1 H, s N*H*), 5.37 (1 H, dd, C*H*), 5.26 (1 H, dd, C*H*), 4.70 (1 H, dd, C*H*), 4.32 (1 H, dd, C*H*) 4.11 (2 H, m, CH_2), 2.98 (2 H, m, CH_2), 2.40 (3 H, s CH_3), 2.35 (2 H, m, CH_2), 2.21 (3 H, s, CH_3), 2.13 (3 H, s, CH_3), 2.09 (3 H, s, CH_3), 2.02 (3 H, s, CH_3), 1.60 (2 H, s, CH_2).

Previously reported compounds were synthesized by following published methods [2-8]

(a)

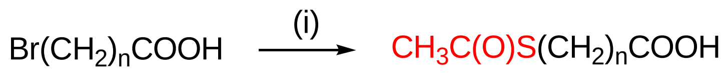

1 **2**

(b)

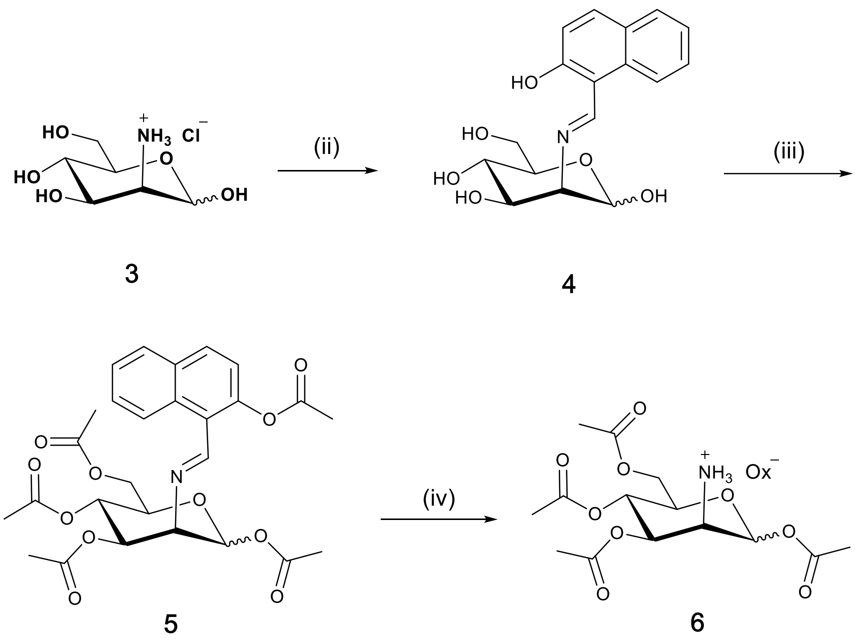

(c)

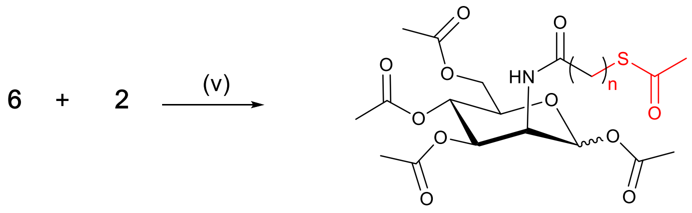

Ac_5ManNTProp: n = 2
Ac_5ManNTBut: n = 3

Scheme S1. **Synthesis of thiol-modified ManNAc analogs**. (**a**) Synthesis of 3-(acetylsulfanyl) propanoic acid (n = 2) and 4-(acetylsulfanyl) butanoic acid (n = 3); conditions: (**i**) potassium thioacetate, acetone, 52%. (**b**) Synthesis of 1,3,4,6-tetra-O-acetyl-2-ammonium-2-deoxy-α-d-mannopyranose oxalate; conditions: (**ii**) sodium acetate, water, 2-hydroxynaphthaldehyde, methanol, room temperature; (**iii**) acetic anhydride, pyridine, 4-(*N*,*N*,-dimethylamino)-pyridine, room temperature, (**iv**) oxalic acid dihydrate, acetone, room temperature (ox- = oxalate). (**c**) Synthesis of Ac_5ManNTProp and Ac_5ManNTBut; conditions: 1-ethyl-3-(3-dimethylaminopropyl) carbodiimide, dimethylformamide, triethylamine, room temperature, 68%.

References

1. Sampathkumar, S. G.; Jones, M. B.; Yarema, K. J., Metabolic expression of thiol-derivatized sialic acids on the cell surface and their quantitative estimation by flow cytometry. *Nat Protoc* **2006,** *1* (4), 1840-51.

2. Kim, E. J.; Sampathkumar, S.-G.; Jones, M. B.; Rhee, J. K.; Baskaran, G.; Yarema, K. J., Characterization of the metabolic flux and apoptotic effects of *O*-hydroxyl- and *N*-acetylmannosamine (ManNAc) analogs in Jurkat (human T-lymphoma-derived) cells. *J Biol Chem* **2004,** *279* (18), 18342-18352.

3. Sampathkumar, S.-G.; Jones, M. B.; Meledeo, M. A.; Campbell, C. T.; Choi, S. S.; Hida, K.; Gomutputra, P.; Sheh, A.; Gilmartin, T.; Head, S. R.; Yarema, K. J., Targeting glycosylation pathways and the cell cycle: sugar- dependent activity of butyrate-carbohydrate cancer prodrugs. *Chem Biol* **2006,** *13* (12), 1265-1275.

4. Sampathkumar, S.-G.; Li, A. V.; Yarema, K. J., Synthesis of non-natural ManNAc analogs for the expression of thiols on cell surface sialic acids. *Nat Protoc* **2006,** *1* (5), 2377-2385.

5. Aich, U.; Campbell, C. T.; Elmouelhi, N.; Weier, C. A.; Sampathkumar, S. G.; Choi, S. S.; Yarema, K. J., Regioisomeric SCFA attachment to hexosamines separates metabolic flux from cytotoxicity and MUC1 suppression. *ACS Chem Biol* **2008,** *3* (4), 230-240.

6. Elmouelhi, N.; Aich, U.; Paruchuri, V. D. P.; Meledeo, M. A.; Campbell, C. T.; Wang, J. J.; Srinivas, R.; Khanna, H. S.; Yarema, K. J., Hexosamine template. A platform for modulating gene expression and for sugar-based drug discovery. *J Med Chem* **2009,** *52* (8), 2515-2530.

7. Du, J.; Che, P.-L.; Aich, U.; Tan, E.; Kim, H. J.; Sampathkumar, S.-G.; Yarema, K. J., Deciphering glycan linkages involved in Jurkat cell interactions with gold-coated nanofibers via sugar-displayed thiols. *Bioorg Med Chem Lett* **2011,** *21* (17), 4980-4984.

8. Almaraz, R. T.; Aich, U.; Khanna, H. S.; Tan, E.; Bhattacharya, R.; Shah, S.; Yarema, K. J., Metabolic oligosaccharide engineering with *N*-acyl functionalized ManNAc analogues: cytotoxicity, metabolic flux, and glycan-display considerations. *Biotechnol Bioeng* **2012,** *109* (4), 992-1006.
